# Formation Mechanism of Thicker Intermetallic Compounds in Friction Stir Weld Joints of Dissimilar AA2024/AZ31B Alloys

**DOI:** 10.3390/ma16010051

**Published:** 2022-12-21

**Authors:** Maoju Tan, ChuanSong Wu, Lei Shi

**Affiliations:** 1 MOE Key Lab for Liquid-Solid Structure Evolution and Materials Processing, Institute of Materials Joining, Shandong University, Jinan 250061, China; 2 Shandong University-Weihai Research Institute of Industrial Technology, Weihai 264209, China

**Keywords:** friction stir welding, Al alloy AA2024, Mg alloy AZ31B, dissimilar alloys, intermetallic compounds

## Abstract

The hybrid structures of AA2024 aluminum alloy and AZ31B magnesium alloy have the advantages of being lightweight, having high specific strength, etc., which are of great application potentials in the aerospace industry. It is a key problem to realize the high-quality welding of these two dissimilar alloys. In this study, the friction stir welding (FSW) tests of AA2024 aluminum alloy and AZ31B magnesium alloy plates of thickness 3 mm were carried out. The intermetallic compounds (IMCs) at the bonding interface were characterized by scanning electron microscope, electron probe, and transmission electron microscope. It was found that the IMCs at the bonding interface in weld nugget zones of dissimilar AA2024/AZ31B FSW has a double-layer structure and a much larger thickness. During the welding process of AA2024/AZ31B, when the boundary of magnesium grains bulges and nucleates, the aluminum atoms diffuse into the magnesium grains, and the γ phase (Al_12_Mg_17_) nucleates at the bonding interface. The β phase (Al_3_Mg_2_) then precipitates at the grain boundary of the γ phase and preferentially grows into γ phase grains. The continuous grain growth to the aluminum side makes the copper contained in AA2024 aluminum alloy concentrate on the side of β phase, which reduces the nucleation work of recrystallization and phase transformation, and further promotes the nucleation and growth of IMCs grains. This is the main reason for the thicker IMCs in the FSW weld of dissimilar AA2024/AZ31B alloys.

## 1. Introduction

With the continuously rising demands of worldwide economic and socially sustainable development requirements for energy saving and emission reduction, lightweight structure design and manufacturing technologies have been increasingly applied in high-speed trains, automobiles, ships, aerospace, and other industrial fields. The use of high-performance light materials such as aluminum alloy and magnesium alloy is an effective way to achieve structural lightweight [1,2]. Among the commonly used aluminum alloys in the aviation industry, the proportion of 2xxx series high-strength aluminum alloys is about 45% [3]. On the other hand, magnesium alloy, as the lightest metallic material, is also increasingly used in the aerospace sector [2,4]. Inevitably, it faces the problem of high-quality welding of composite components composed of 2xxx series aluminum alloy and magnesium alloy. Because the physical and chemical properties of these two alloys are quite different, a large amount of brittle intermetallic compounds (IMCs) and porosities are easily generated in the dissimilar welds by traditional welding processes [1,5,6], and the strength of the joint is very low, which cannot meet the application requirements. Friction Stir Welding (FSW), as a solid-state welding process, has been proven to be the first choice for high-quality welding of aluminum/magnesium (Al/Mg) dissimilar alloys because of its low welding temperature, large plastic deformation, and high strain rate [1,2,5].

When Al/Mg dissimilar alloys are joined by the FSW process, the friction heat and plastic deformation induced by the tool leads to materials softening, intermixing, and flowing around the tool so that a solid weld with fine and equiaxed recrystallized microstructure is obtained, but the intermetallic compounds (IMCs) produced in the weld is still the main factor affecting the joint strength [1,2]. The hardness and brittleness of IMCs can not only reduce the strength of the joint but also be the main factor affecting the fracture toughness of the joint [7,8,9]. Some researchers have studied the formation mechanism of IMCs in dissimilar FSW of Al/Mg alloys and found that IMCs were formed by solid diffusion mainly or local constitutional liquation at some conditions [9,10,11,12]. It is not clear whether there are other generation mechanisms, such as forming an amorphous layer on the interface without undesirable IMC, as in the case of Al/Fe joining [13]. Therefore, the formation mechanism of IMCs in Al/Mg FSW still needs further study.

A great deal of experimental research on Al/Mg dissimilar alloy FSW has been carried out, but most researchers choose 6xxx series Al alloy [14,15,16,17,18,19,20] joined to AZ31B Mg alloy, with a small number of researchers choosing 5xxx or 7xxx series Al alloy [20,21]. There are relatively few FSW experimental studies on 2xxx series high-strength Al alloy and AZ31B Mg alloy. Khodir et al. [22] carried out the butt FSW test of 2024-T3 Al alloy and AZ31 Mg alloy plates of 3 mm thickness and studied the effect of welding speed on the macro-morphology and microstructure of the dissimilar joint. They found that there were a lot of intermetallic compounds in the irregular layered structure in the stirring zone. Song et al. [23] measured the tensile strength of the FSW joint of 2024-T3/AZ31B, which was higher than that of the fusion welded joint but still lower than 70 MPa. The reason for the low joint strength is that a thick intermetallic compound layer is easily formed in the FSW weld of 2024-T3/AZ31B. However, why is it easy to form a thick intermetallic compound layer in the FSW weld of AA2024/AZ31B? What are the characteristics of the morphology, distribution, and formation mechanism of thicker intermetallic compounds in dissimilar FSW joints? In this study, we used SEM (Scanning Electron Microscope), EPMA (electron probe microanalysis), and TEM (transmission electron microscope) to characterize the intermetallic compounds produced in the weld in detail. We observed the thickness and distribution of intermetallic compounds in the FSW weld of 2024-T3/AZ31B and discussed the important factors influencing the above problems.

## 2. Experimental

**Figure 1:**
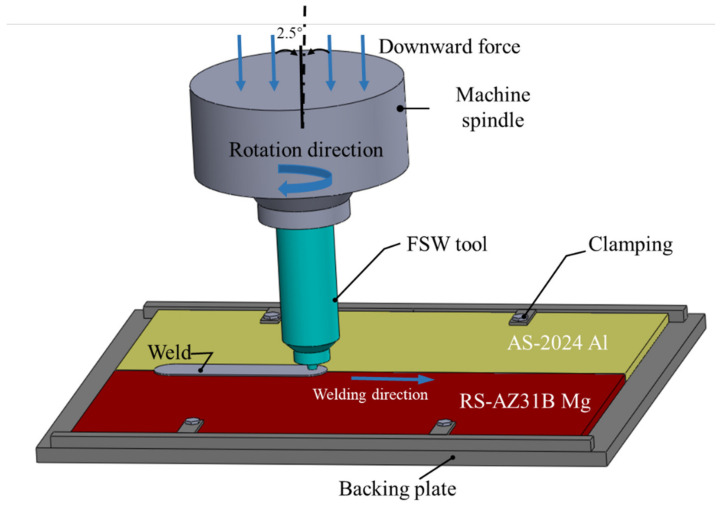
Schematic diagrams of Al/Mg FSW experimental set-up.

**Figure 2:**
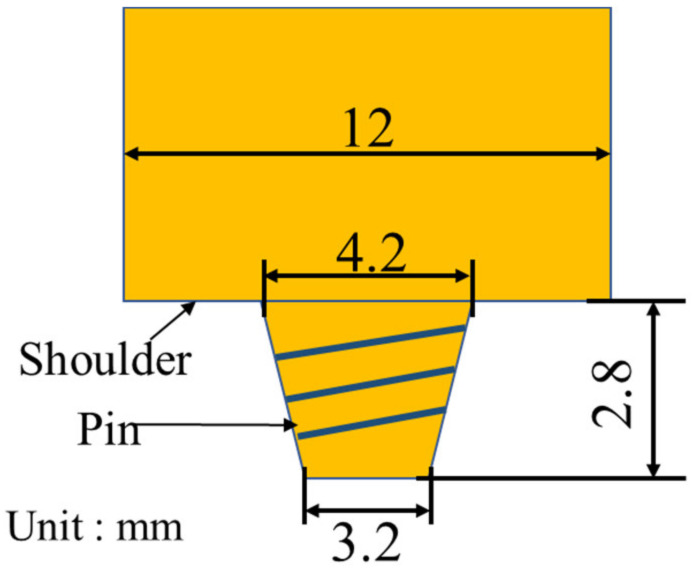
Schematic of FSW tool.

In this study, Al alloy AA2024-T4 and Mg alloy AZ31B-H24 plates with dimensions of 3 mm thickness, 200 mm length, and 65 mm width were butt welded, as shown in Figure 1. The mass spectrometer JSM-7800f was used to determine the chemical composition of the base plates. Their chemical compositions and mechanical properties are listed in Table 1. The H13 steel tool was with a concave shoulder of 12 mm diameter, a threaded pin of 2.8 mm length, a root diameter of 4.2 mm, and a tip diameter of 3.2 mm, as shown in Figure 2. According to the weld formation of preliminary tests, when the Al alloy is placed on the advanced side (AS), it is easy to obtain high-strength welds. Therefore, in this study, AA2024-T4 was placed on AS while the AZ31B-H24 plate was on the retreating side (RS). The offset of the tool was zero. The optimized process parameters were as follows: tool rotation speed of 700 rpm, welding speed of 40 mm/min, tool tilt angle of 2.5°, and plunge depth of shoulder 0.1 mm.

After welding, the specimens at the transverse cross-section of welds were cut with the electric spark wire cutting machine. The specimens were then processed and polished with emery papers and 0.5 mm diamond slurry. For the microstructure characterization, the samples were etched by Keller reagent with 1.0 mL HF + 1.5 mL HCl + 2.5 mL HNO3 + 95 mL distilled water for 5 s and by picric acid solution for 10 s. Optical microscope and scanning electron microscopy (SEM) observation and characterization were made at the transverse cross-section of dissimilar welds. For transmission electron microscope (TEM) and scanning TEM (STEM) characterization, the samples were cut by a focused ion beam (FIB). Electron probe microanalysis (EPMA) was also conducted at a specific position.

## 3. Results and Discussion

### 3.1. SEM Images of IMCs

**Figure 3:**
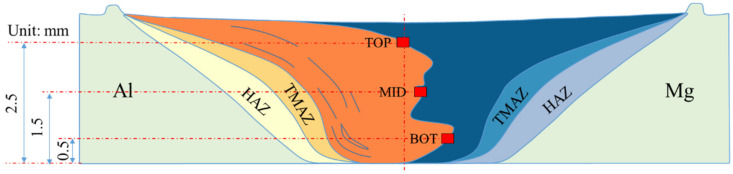
Schematic illustration of weld cross section and SEM characterization locations: TMAZ-thermo-mechanically affected zone, HAZ- heat affected zone. TOP, MID, and BOT represent the top, middle, and bottom regions of the weld, respectively.

**Figure 4:**
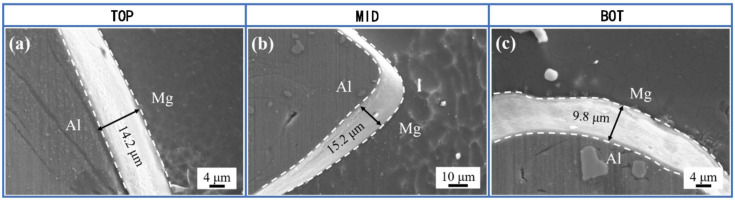
SEM images at three locations in FSW weld: (**a**) location TOP, (**b**) location MID, (**c**) location BOT.

As schematically illustrated in Figure 3, three positions located at the top, middle and bottom regions in the weld nugget zone (WNZ) at the transverse cross-section of a dissimilar weld were selected for SEM characterization. Figure 4 shows the SEM images of intermetallic compounds (IMCs) at TOP, MID, and BOT, and the IMCs thickness was 14.2 µm, 15.2 µm, and 9.8 µm, respectively. When compared to those in the AA6061/AZ31B joint with corresponding data of 1.6 µm, 7.7 µm, and 1.7 µm [24,25], the IMCs thickness in the AA2024/AZ31B joint is much larger.

Using the temperature detection method proposed in [26], we also conducted temperature measurements. We obtained a peak temperature in FSW of 373.5 °C. Under almost the same welding process conditions, the temperature measurement results show that the welding temperature in AA2024/AZ31B FSW is not much different from that in the 6061/AZ31B case (about 360~380 °C) [26,27], and neither of them reaches the eutectic temperature (437 °C or 450 °C) of Al/Mg binary alloy system. However, the thickness of IMCs in AA2024/AZ31B weld is much larger, so it is necessary to find other reasons besides the different welding temperatures. The biggest difference between AA2024 and AA6061 lies in the difference in alloy element content, and AA2024 aluminum alloy contains more Cu elements, as listed in Table 1. Perhaps the strengthening element Cu is the key factor in determining the thickness of the IMCs layer in the AA2024/AZ31B weld.

### 3.2. Bonding Interface

Electron probe microanalysis (EPMA) was carried out to characterize the distribution of elements at the interface. Since the intermetallic compound is thick in the upper and middle parts of the weld nugget zone while the size of the FIB sample is about 6 μm × 7 μm, the observation location was selected at BOT in Figure 3, the lower part of the weld where the intermetallic compound is relatively thin. This can cover the aluminum/magnesium matrix as much as possible on the basis of photographing the intermetallic compound.

**Figure 5:**
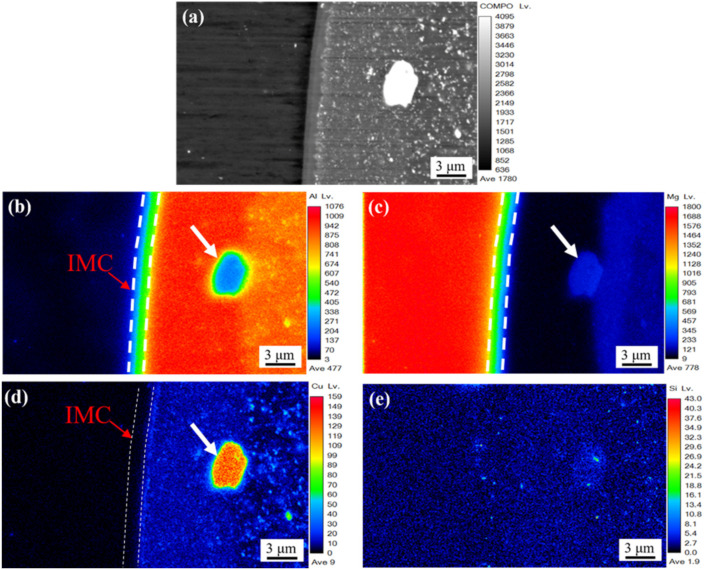
EPMA scanning at location BOT (**a**), distribution map of Al (**b**), Mg (**c**), Cu (**d**), and Si (**e**).

Figure 5 presents the EPMA measurement result on Al/Mg bonding interface in the FSW weld. It can be seen from Figure 5b and c that there is a composition transition of aluminum and magnesium in the intermetallic compound layer, which indicates the existence of double-layer IMCs. However, it is difficult to find this in the SEM image in Figure 4. Figure 5d shows that a large amount of copper exists in the strengthening phase S-phase Al_2_CuMg, as shown by the white arrow in the figure. The element segregation of copper gathers at the boundary of the IMC layer, forming a fine Cu strip. It can be seen from Figure 5e that the Si element mainly exists in the position of small-sized reinforced phase particles of AA2024 aluminum alloy, and it is generally characterized by dispersion distribution.

**Figure 6:**
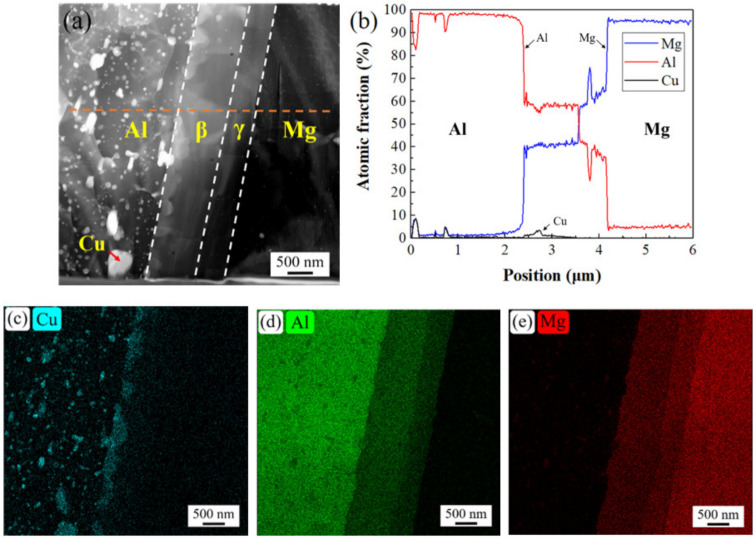
STEM image at location BOT (**a**), line scan results (**b**), element distribution of Cu (**c**), Al (**d**), and Mg (**e**).

Figure 6 shows the results of STEM surface scanning and line scanning at location BOT in the AA2024/AZ31B FSW weld specimen. Figure 6a is a dark field image. It can be seen that the intermetallic compound layer has a double-layer structure and its grain size is large. The strengthening phase mainly exists in aluminum alloy, which is white granular in the image. Figure 6b is the line scanning result along the dotted orange line in Figure 6a. According to the atomic ratio of aluminum/magnesium element, it can be determined that the intermetallic compound of the bilayer is β phase (Al_3_Mg_2_) and γ phase (Al_12_Mg_17_), respectively. In Figure 6b, there is a small error in the aluminum/magnesium atomic ratio at the position of Al_12_Mg_17_. This is because the defect position of magnesium alloy in Figure 6a was scanned at this position. It may be that the height of this position was inconsistent with other positions during the FIB cutting process, and then finally scanned aluminum/magnesium atomic ratio has an error at this position. Figure 6c–e are plane scanning images of Cu, Al, and Mg elements, respectively. It can be seen that there is a stepped transition between magnesium and aluminum, which is consistent with the results of the electron probe test. At the same time, it can be seen that there is segregation and aggregation of Cu, which is located between Al_3_Mg_2_ and Al matrix, and Cu exists in the form of a smaller strengthening phase.

**Figure 7:**
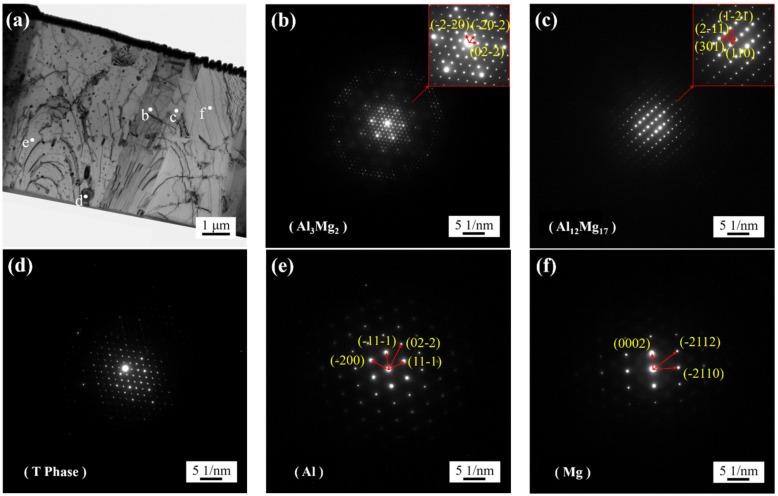
TEM diffraction pattern at location BOT: (**a**) diffraction location (corresponding to Figure 4a), and (**b**–**f**) diffraction pattern at locations b–f in (**a**).

Figure 7 shows the diffraction pattern of the FSW joint. Figure 7b–f, respectively, show the diffraction patterns at the locations of the white marks in Figure 7a. By calculating the interplanar spacing and angle and comparing with the standard diffraction pattern, it can be determined that Figure 7b corresponds to Al_3_Mg_2_ with face-centered cubic structure, Figure 7c to Al_12_Mg_17_ with body-centered cubic structure, Figure 7e to Al with face-centered cubic structure, and Figure 7f to Mg with close-packed hexagonal structure. Figure 7d shows the diffraction pattern of the strengthening phase T-phase.

According to the calibration of diffraction spots of transmission electron microscope samples, the types of two intermetallic compounds are determined, namely Al_3_Mg_2_ with a face-centered cubic structure and Al_12_Mg_17_ with a body-centered cubic structure. The grain size in IMCs is large, and the diffraction patterns are single crystal diffraction results, which are consistent with the scanning results of STEM. The strengthening phases are mainly the large particles in AA2024 aluminum alloy, namely, S-phase Al_2_CuMg and relatively small-sized T-phase particles. The uneven distribution of the post-welding strengthening phase will affect the strength of welded joints, which may also be one of the reasons for the low strength of welded joints. At the same time, fine strengthening phase particles are clustered on one side of the Al_3_Mg_2_ phase, which will promote nucleation during the recrystallization of intermetallic compound grains. The size of the agglomerated strengthening phase is relatively small, while the size of the S-phase Al_2_CuMg is generally relatively large. This may be because the S-phase is involved in the formation of intermetallic compounds and the transformation has taken place. T-phase particles are relatively stable and will not change easily. It is speculated that the strengthening phase of segregation is T-phase. Although T-phase particles can pin the grain boundary in aluminum alloy and improve the strength of AA2024 aluminum alloy, the uneven distribution of T-phase particles caused by segregation will reduce the strength of welded joints [28]. A large number of strengthening phase particles exist on one side of the Al_3_Mg_2_ phase, which will significantly reduce the nucleation work of recrystallization and phase deformation of intermetallic compounds, greatly promote the nucleation and growth of grains, and finally increase the thickness of intermetallic compounds.

### 3.3. Formation Mechanism of Thicker IMCs

According to the TEM observation results, the grains of intermetallic compounds grow mainly from the side of Mg alloy to the side of Al alloy, and the position of β phase (Al_3_Mg_2_) occupies the former Al matrix, which pushes the Cu strengthening phase to the side of β phase during the grain growth, and the formation time of Al_3_Mg_2_ is no earlier than Al_12_Mg_17_.

**Figure 8:**
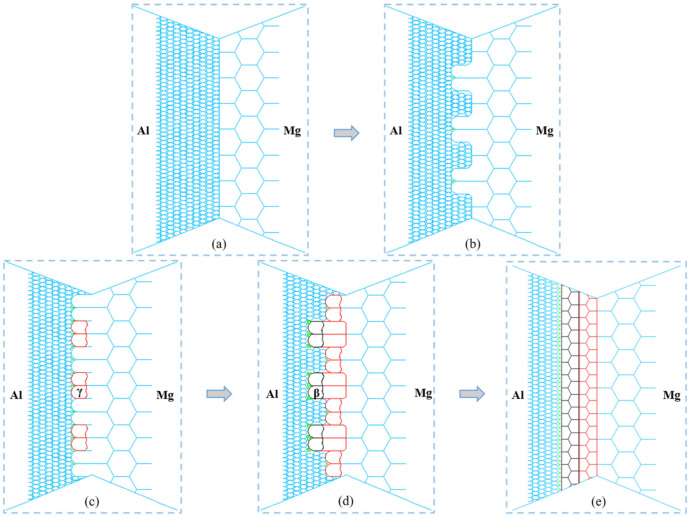
Schematic of IMCs formation at the bonding interface of AA2024/AZ31B FSW weld: (**a**) before welding, (**b**) first stage of welding;(**b**) second stage of welding, (**c**) third stage of welding, (**d**) fourth stage of welding, and (**e**) completion of welding.

Figure 8 schematically shows the formation process of intermetallic compounds at the bonding interface in AA2024/AZ31B FSW, which belongs to the atomic diffusion mechanism. Because the Al/Mg interface during the FSW process is rough at the micro-scale, the recrystallization nucleation and growth of Al alloy grains and Mg alloy grains will first occur at elevated temperatures. As this process continues, the Al/Mg grains come into contact, and the Al/Mg interface becomes obvious, accompanied by intense atomic diffusion.

EBSD test results show that the grain size of Mg alloy (~2.77 μm) in WNZ is much larger than that of Al alloy (~0.73 μm). On this premise, because the deformation degree of grains on both sides of the Al/Mg interface is different, the grain size of Al alloy is smaller, and the dislocation density will be much higher than that of Mg alloy. Therefore, the subgrain formed in Al alloy grains after multilateralization is smaller than that in Mg alloy grains. In this case, at the Al/Mg interface, some sub-grains of Mg grains will protrude into the Al grains through the migration of the Al/Mg interface and grow by consuming the sub-grains in the Al grains so that the free energy of the system will decrease, thus forming a nucleation core of recrystallization, as shown in Figure 7b. This nucleation mode belongs to grain boundary bulge nucleation [29,30]. As the grain boundary of Mg alloy bulges and grows, the specific surface area of the bulged part of the grain increases, and the diffusion of Al atoms into Mg grains will be more intense. At the same time, recrystallization grain growth will occur in both Al and Mg grains. The continuous growth of Mg grains will make the strengthening phase in the Al matrix gather at the grain boundary, which will promote the nucleation of grains.

At the Mg grain boundary near Al grain, it belongs to the high-angle grain boundary, and its surface energy is high, which can reduce nucleation work. Moreover, there are many defects, such as the dislocation of grain boundary in this position. In addition, the grain boundary is prone to the segregation of components, so the required driving force for phase transformation here is low, and phase transformation nuclei are more likely to occur. Once the thermal activation energy reaches the grain boundary formation energy of phase transformation, phase transformation nuclei will occur, and the growth of nuclei will be realized by the diffusion of atoms. There is no obvious liquefaction phenomenon in intermetallic compounds, and its formation probably belongs to the diffusion phase transition. As the protruding part of the Mg grain is diffused by more Al atoms, it is easy to reach the atomic ratio required by γ phase Al_12_Mg_17_. There are more Al atoms in the grains of Mg, and the newly formed γ phase Al_12_Mg_17_ will preferentially grow into the grains of Mg. In the process of phase transformation, the geometric dynamic recrystallization of Mg grains will occur at the same time, protruding to the grain boundaries of Al grains, as shown in Figure 8c.

In the process of continuous growth of γ-phase Al_12_Mg_17_ into Mg grains, when it consumes enough Al atoms in Mg grains, it will stop this phase transformation mode with less energy consumption. At the same time, at elevated temperatures, γ-phase will not only grow up through the diffusion of atoms but also grow out into the Al matrix, and Al atoms will diffuse into γ-phase grains violently. According to the binary phase diagram of Al-Mg [12], with the decrease in temperature, part of the β phase will precipitate at the grain boundary of the γ phase. Because there are many defects at the grain boundary of the γ phase near the Al side, at the same time, more strengthening phase particles will promote the phase transformation nucleus, and it is easier to reach the grain boundary formation energy of phase transformation so that the phase transformation nucleus will occur. Because more Al atoms diffuse into the protruding part of the γ-phase grain, it is easier to reach the atomic ratio required by β-phase Al_3_Mg_2_, and there are more Al atoms in the γ-phase grain, so the newly formed β-phase Al_3_Mg_2_ will preferentially grow into the γ-phase grain. At the same time, the γ-phase is transformed into the β-phase, and the γ-phase in other positions still bulges and nucleates at grain boundaries, as shown in Figure 8d. During the continuous growth of intermetallic compounds to the Al side, the particles of the strengthening phase will be squeezed to the side of the β phase continuously, which leads to a large aggregation of strengthening phase particles between the β phase and Al matrix. This will promote the nucleation of the β phase and facilitate the growth of intermetallic compounds.

As more and more γ-phase is transformed into β-phase, this phase transformation mode with less energy consumption will stop when enough Al atoms in γ-phase grains are consumed in this process. The strengthening phase, S-phase Al_2_CuMg in AA2024 alloy, is unstable at elevated temperatures. It is very likely that the Mg atoms in S-phase will be utilized by the β-phase in the process of grain boundary migration. In this process, S-phase may participate in the formation of intermetallic compounds. This can also explain why there is no S phase with large particles in the reinforced phase of segregation at the boundary, but all T phases with small sizes. Because the transition from the γ phase to the β phase is accompanied by the migration of interface, Al and Mg atoms constantly move into a new phase, the interface moves to the γ phase, and the new phase grows up. The final condition is that the energy gradient of atom diffusion in the γ phase and β phase is the same, so the final interface between the γ phase and β phase is usually flat. The continuous phase transformation of γ phase and β phase and the process of recrystallization grain growth make the position and angle of a grain boundary change and finally tend to be stable. However, the continuous growth of grains to the Al side causes the strengthening phase to be biased to the side of the β phase, as shown in Figure 8e. This is the speculated mechanism of diffusion formation of intermetallic compounds at high temperatures/high strain rates in the AA2024/AZ31B FSW process.

## 4. Conclusions

(1) The SEM images of intermetallic compounds at the interface show that the thickness of IMCs is larger in dissimilar FSW of AA2024/AZ31B than that in dissimilar FSW of AA6061/AZ31B. Because the welding temperature in AA2024/AZ31B FSW is very close to that in AA6061/AZ31B FSW under the same conditions, it is clear that the larger IMCs thickness in AA2024/AZ31B weld is caused by the copper content in AA2024.
(2) The EPMA and STEM images show that there is an obvious gradient transition of aluminum and magnesium in intermetallic compounds, which proves that intermetallic compounds have a double-layer structure of β phase (Al_3_Mg_2_) and γ phase (Al_12_Mg_17_). Copper was aggregated between the intermetallic compound and aluminum matrix, and segregated between β phase and aluminum matrix in the form of a strengthening phase.
(3) In the dynamic recrystallization process during AA2024/AZ31B FSW, after the grain boundaries of Mg grains bulge and nucleate, the Al atoms quickly diffuse into Mg grains. The γ phase nucleates at the grain boundary and its growth rate into the original Mg grain is higher than that into the Al grain. The grain boundary bulge nucleation and phase transformation nucleation of Mg grain occur at the same time. The grain boundary of γ-phase bulges and nucleates. Then, the β-phase precipitates at the grain boundary of the γ-phase and preferentially grow into the original γ-phase grain. The continuous grain growth to the Al side makes the strengthening phase Cu bias to the side of the β phase. The recrystallization grain growth process of γ phase and β phase makes some changes in grain boundary position and angle and finally tends to be stable.

## Figures and Tables

**Table 1:** Nominal composition and mechanical properties of base materials.

Alloy	Nominal Chemical Composition (wt.%)									Mechanical Properties	
	Si	Fe	Cu	Mn	Mg	Cr	Ti	Zn	Al	UTS(MPa)	EL (%)
2024-T4	0.252	0.158	4.44	0.651	1.37	0.13	-	0.094	Bal.	462	15
AZ31B	0.034	0.005	0.004	0.322	Bal.	-	-	0.936	3.91	251	11

## Data Availability

Data sharing is not applicable.

## References

[1] Wu C.S., Lv X.Q., Su H., Shi L. (2022). Research progress in dissimilar friction stir welding of Aluminium/Magnesium alloys. J. Mech. Eng..

[2] Shah L.H., Othman N.H., Gerlich A. (2018). Review of research progress on aluminium–magnesium dissimilar friction stir welding. Sci. Technol. Weld. Join..

[3] Wang J.G., Wang Z.T. (2013). Progress in deformation aluminium alloys for aeronautic & astronautics industry. Light Alloy. Fabr. Technol..

[4] Zhong H., Liu P.Y., Zhou T.H. (2002). Application and prospects of Magnesium and its alloys in aerospace. Aviat. Maint. Eng..

[5] Ma Z.Y., Shang Q., Ni D.R., Xiao B.L. (2018). Friction stir welding of Magnisum alloys: A Review. Acta Metall. Sin..

[6] Sachin K., Wu C.S. (2017). Review: Mg and Its Alloy—Scope, future perspectives and recent advancements in welding and processing. J. Harbin Inst. Technol..

[7] Liang Z., Chen K., Wang X., Yao J., Yang Q., Zhang L., Shan A. (2013). Effect of Tool Offset and Tool Rotational Speed on Enhancing Mechanical Property of Al/Mg Dissimilar FSW Joints. Met. Mater. Trans. A.

[8] Fu B., Qin G., Li F., Meng X., Zhang J., Wu C. (2014). Friction stir welding process of dissimilar metals of 6061-T6 aluminum alloy to AZ31B magnesium alloy. J. Mater. Process. Technol..

[9] Yamamoto N., Liao J., Watanabe S., Nakata K. (2009). Effect of Intermetallic Compound Layer on Tensile Strength of Dissimilar Friction-Stir Weld of a High Strength Mg Alloy and Al Alloy. Mater. Trans..

[10] Rao H.M., Ghaffari B., Yuan W., Jordon J., Badarinarayan H. (2016). Effect of process parameters on microstructure and mechanical behaviors of friction stir linear welded aluminum to magnesium. Mater. Sci. Eng. A.

[11] Verma J., Taiwade R.V., Sapate S.G., Patil A.P., Dhoble A.S. (2017). Evaluation of Microstructure, Mechanical Properties and Corrosion Resistance of Friction Stir-Welded Aluminum and Magnesium Dissimilar Alloys. J. Mater. Eng. Perform..

[12] Firouzdor V., Kou S. (2010). Formation of Liquid and Intermetallics in Al-to-Mg Friction Stir Welding. Met. Mater. Trans. A.

[13] Liu F., Dong P., Zhang J., Lu W., Taub A., Sun K. (2020). Alloy amorphization through nanoscale shear localization at Al-Fe interface. Mater. Today Phys..

[14] Zhao Y., Lu Z., Yan K., Huang L. (2015). Microstructural characterizations and mechanical properties in underwater friction stir welding of aluminum and magnesium dissimilar alloys. Mater. Des..

[15] Sato Y.S., Park S.H.C., Michiuchi M., Kokawa H. (2004). Constitutional liquation during dissimilar friction stir welding of Al and Mg alloys. Scr. Mater..

[16] Mofid M.A., Abdollah-Zadeh A., Ghaini F.M. (2012). The effect of water cooling during dissimilar friction stir welding of Al alloy to Mg alloy. Mater. Des..

[17] Chang W.-S., Rajesh S., Chun C.-K., Kim H.-J. (2011). Microstructure and Mechanical Properties of Hybrid Laser-Friction Stir Welding between AA6061-T6 Al Alloy and AZ31 Mg Alloy. J. Mater. Sci. Technol..

[18] Ji S., Huang R., Meng X., Zhang L., Huang Y. (2017). Enhancing Friction Stir Weldability of 6061-T6 Al and AZ31B Mg Alloys Assisted by External Non-rotational Shoulder. J. Mater. Eng. Perform..

[19] Kumar S., Wu C. (2020). Suppression of intermetallic reaction layer by ultrasonic assistance during friction stir welding of Al and Mg based alloys. J. Alloy. Compd..

[20] Kwon Y.J., Shigematsu I., Saito N. (2008). Dissimilar friction stir welding between magnesium and aluminum alloys. Mater. Lett..

[21] Kang J., Fu R.D., Luan G.H., He M. (2011). Effect of the offseting on microstructures and mechanical properties of FSW joints of 7075 Al alloys-AZ31B Mg alloys. Rare Met. Technol..

[22] Khodir S.A., Shibayanagi T. (2007). Dissimilar Friction Stir Welded Joints between 2024-T3 Aluminum Alloy and AZ31 Magnesium Alloy. Mater. Trans..

[23] Song B. (2017). Study on Material Flow and Joint Property of the Dissimialr Friction Stir Joining for Mg and Al Alloys.

[24] Lv X., Wu C., Yang C., Padhy G. (2018). Weld microstructure and mechanical properties in ultrasonic enhanced friction stir welding of Al alloy to Mg alloy. J. Mater. Process. Technol..

[25] Zhao J., Wu C.S., Su H. (2020). Ultrasonic effect on thickness variations of intermetallic compound layers in friction stir welding of aluminium/magnesium alloys. J. Manuf. Process..

[26] Lv X.Q., Wu C.S., Sun Z. (2022). Effects of ultrasonic vibration on material flow and thermal cycles in friction stir welding of dissimilar Al/Mg alloys. Metall. Mater. Trans. A.

[27] Zhao J., Wu C., Su H. (2022). Ultrasonic vibration-induced thinning of intermetallic compound layers in friction stir welding of dissimilar Al/Mg alloys. Sci. Technol. Weld. Join..

[28] Tan M.J., Wu C.S., Su H. (2022). Ultrasonic effects on microstructures and mechanical properties of friction stir weld joints of dissimilar AA2024 /AZ31B alloys. Weld. World.

[29] Yu P., Wu C., Shi L. (2021). Analysis and characterization of dynamic recrystallization and grain structure evolution in friction stir welding of aluminum plates. Acta Mater..

[30] Huang K., Logé R.E. (2016). A review of dynamR.E. ic recrystallization phenomena in metallic materials. Mater. Des..

